# Engineering macrophage phenotype switching via nucleotide‐binding oligomerization domain‐like receptor protein 3 inflammasome inhibition: A translational approach using antibiotic cement for diabetic foot ulcers

**DOI:** 10.1002/btm2.70073

**Published:** 2025-10-15

**Authors:** Yi Zhang, Fusen Jia, Ming Li, Xin Tang, Fei Yang

**Affiliations:** ^1^ Department of Hand and Foot Surgery Zibo Central Hospital Zibo China; ^2^ Department of Orthopedics First Affiliated Hospital of Dalian Medical University Dalian China; ^3^ Department of Joint Surgery Zibo Lianchi Orthopedic Hospital Zibo China; ^4^ Department of Orthopedics Zibo Central Hospital Zibo China

**Keywords:** antibiotic‐loaded bone cement, diabetic foot ulcer, macrophage polarization, NLRP3 inflammasome, translational immunomodulation

## Abstract

Diabetic foot ulcers (DFUs), a debilitating complication of diabetes, are exacerbated by persistent inflammation that disrupts wound repair. This study explores the therapeutic potential of antibiotic‐loaded bone cement (ALBC) in modulating NLRP3 inflammasome activation and macrophage polarization to resolve chronic inflammation and accelerate healing. Using db/db diabetic mice with dorsal wounds and RAW264.7 macrophages under high‐glucose conditions, we tested graded ALBC doses (high‐dose ALBC, low‐dose ALBC, and medium‐dose ALBC) both in vivo and in vitro. Multi‐modal analyses—including cytokine profiling (enzyme‐linked immunosorbent assay), macrophage phenotyping (flow cytometry/immunofluorescence), and molecular pathway interrogation (reverse transcription quantitative PCR/Western blot)—revealed that ALBC dose‐dependently suppressed NLRP3 inflammasome assembly, reduced IL‐1β/IL‐18 secretion, and skewed macrophages toward anti‐inflammatory M2 phenotypes. Pharmacological NLRP3 activation reversed these effects, confirming pathway specificity. ALBC‐treated wounds exhibited accelerated re‐epithelialization, collagen deposition, and angiogenesis, correlating with attenuated systemic inflammation. Crucially, clinical DFU samples mirrored preclinical findings, showing NLRP3 downregulation and M2 dominance in ALBC‐responsive cases. These results demonstrate that ALBC orchestrates immunometabolic reprogramming by silencing NLRP3‐driven inflammation and fostering pro‐reparative macrophage responses. By bridging biomaterial engineering with immunomodulation, this work advances a translatable strategy for refractory DFU management, offering a dual‐action therapeutic platform that combines localized antibiotic delivery with microenvironmental immune reset.

**Abbreviations**
AGEs: advanced glycation end‐products
ALBC: antibiotic‐loaded bone cement
ALBC‐H: high‐dose antibiotic‐loaded bone cement
ALBC‐L: low‐dose antibiotic‐loaded bone cement
ALBC‐M: medium‐dose antibiotic‐loaded bone cement
ANOVA: analysis of variance
ASC: apoptosis‐associated speck‐like protein containing a CARD
ATP: adenosine triphosphate
BCA: bicinchoninic acid (protein assay)
CARD: caspase recruitment domain
cDNA: complementary DNA
DAPI: 4′,6‐diamidino‐2‐phenylindole
DFUs: diabetic foot ulcers
ELISA: enzyme‐linked immunosorbent assay
FDA: U.S. Food and Drug Administration
GAPDH: glyceraldehyde‐3‐phosphate dehydrogenase
H&E: hematoxylin and eosin
HG: high‐glucose
IF: immunofluorescence
IHC: immunohistochemistry
IL‐1β (IL‐1B)): interleukin‐1 beta
IL‐1β/IL‐18: interleukin‐1 beta / interleukin‐18
iNOS: inducible nitric oxide synthase
LPS: lipopolysaccharide
NF‐κB (NF‐KB): nuclear factor kappa‐light‐chain‐enhancer of activated B cells
PBS: phosphate‐buffered saline
PCR: polymerase chain reaction
PMMA: polymethyl methacrylate
PRP: platelet‐rich plasma
PVDF: polyvinylidene difluoride
RIPA: radioimmunoprecipitation assay (lysis buffer)
RT‐qPCR: reverse transcription quantitative PCR
SD: standard deviation
SDS‐PAGE: sodium dodecyl sulfate–polyacrylamide gel electrophoresis
STAT6: signal transducer and activator of transcription 6
TBST: tris‐buffered saline with tween‐20
TLRs: toll‐like receptors
TMB: 3,3′,5,5′‐tetramethylbenzidine
TNF‐α/TNF‐a: tumor necrosis factor‐alpha
TNF‐β: tumor necrosis factor‐beta
VEGF: vascular endothelial growth factor

**Translational Impact Statement**

This study bridges biomaterial engineering with immunomodulatory therapy by repurposing antibiotic‐loaded bone cement—a clinically established orthopedic material—into a dual‐action platform for diabetic foot ulcers (DFU). We demonstrate its capacity to locally suppress NLRP3‐driven inflammation while reprogramming macrophages toward pro‐healing phenotypes, validated across murine models and human clinical specimens. By leveraging an FDA‐approved material with inherent antibiotic delivery, our approach offers an immediately deployable strategy to resolve chronic inflammation and restore wound healing trajectories in DFU patients, addressing a critical gap in current wound care paradigms through engineering‐augmented immune reset.

## 1 INTRODUCTION

Diabetic foot ulcers (DFUs) are among the most common and disabling high‐risk complications of diabetes,^1^ posing a major challenge to public health systems worldwide. In 2019, the global prevalence of diabetes was estimated at 9% (463 million adults), with a lifetime incidence of foot ulcers reaching up to 25%.^2^, ^3^, ^4^ Risk factors for delayed ulcer healing include poor glycemic control, peripheral neuropathy, peripheral vascular disease, and immune suppression, with up to 85% of DFUs attributed to neuropathy‐induced alterations in foot pressure.^5^ Owing to factors such as long disease duration, foot deformities, and callus formation,^6^ approximately 40% of patients experience ulcer recurrence within 1 year of healing, with recurrence rates increasing to 60% and 65% at 3 and 5 years, respectively.^4^ A history of DFUs is therefore the strongest predictor of secondary ulcer development, which not only impairs patients' quality of life but also increases healthcare burdens and raises the risk of amputation due to infection and ischemia.^7^, ^8^ With the global diabetic population continuously increasing and the lack of effective therapies, there is an urgent need for novel wound‐healing strategies.^9^, ^10^ Current clinical treatments—such as surgical debridement, hyperbaric oxygen therapy, and negative‐pressure wound therapy—can provide partial benefits, while emerging nanomedicine and stem cell therapies^11^, ^12^ offer new hope but remain limited by technical uncertainties and inability to fully address chronic inflammation and delayed repair.^11^ Thus, developing therapeutic approaches capable of effectively modulating local inflammation and promoting tissue regeneration has become a priority in DFU research.^13^, ^14^

As essential immune system effector cells, macrophages play a central regulatory role in chronic wound healing.^15^ In response to stimuli from the local microenvironment, macrophages can differentiate into distinct functional phenotypes, primarily pro‐inflammatory M1 and pro‐repair M2 macrophages.^16^ M1 macrophages, during the early stages of infection, clear pathogens by secreting inflammatory cytokines such as TNF‐α and IL‐1β.^17^ However, their prolonged activation may aggravate tissue damage and impair wound healing. In contrast, M2 macrophages promote angiogenesis, basement membrane reconstruction, and cell proliferation by producing anti‐inflammatory and reparative cytokines such as IL‐10 and TGF‐β.^18^, ^19^ Therefore, in DFUs—characterized by chronic inflammation—inducing macrophage polarization from the M1 to the M2 phenotype may serve as an important strategy to alleviate inflammation and accelerate tissue repair. Based on this rationale, the present study focused on macrophage polarization as a regulatory target to explore novel therapeutic interventions.

The NLRP3 inflammasome, as a key inflammatory signaling complex, was a central mediator in the inflammatory cascades associated with various chronic diseases. It recruited apoptosis‐associated speck‐like protein containing a CARD (ASC) and activated Caspase‐1, thereby promoting the maturation and release of IL‐1β and IL‐18,^20^ continuously amplifying the inflammatory response. In patients with DFUs, prolonged exposure to high‐glucose (HG) conditions, oxidative stress, and the accumulation of advanced glycation end‐products (AGEs) led to sustained NLRP3 expression, resulting in an imbalanced inflammatory microenvironment and impaired tissue repair.^21^ Previous studies suggested that inhibitors targeting the NLRP3 inflammasome, such as MCC950, could improve chronic wound healing; however, no standardized treatment protocol had yet been established.^22^ Antibiotic‐loaded bone cement (ALBC), developed initially as a local sustained‐release system for bone defect repair, had recently been applied to the treatment of various chronic refractory wounds, including DFUs, with certain clinical efficacy.^23^, ^24^ Nevertheless, the specific immunomodulatory mechanisms of ALBC remained poorly understood, particularly whether it promoted wound healing by modulating the NLRP3 inflammasome pathway.

Although current therapeutic options—such as systemic antibiotics, corticosteroids, negative‐pressure wound therapy, and platelet‐rich plasma (PRP)—could partially alleviate infection and improve microcirculation, they remained insufficient in regulating the immune microenvironment and promoting cellular regeneration.^25^, ^26^ Several clinical observations indicated that even under well‐controlled infection, DFUs still exhibited slow healing and high recurrence rates.^27^ Therefore, developing novel local therapeutic materials with anti‐inflammatory and pro‐repair functions held significant research and clinical value. As a material platform with controlled‐release properties, ALBC has already demonstrated promising results in infection control and tissue support.^28^ Based on these advantages, the present study further proposed that ALBC might exert deeper wound‐healing effects by regulating immune responses—particularly via the NLRP3 inflammasome and macrophage polarization pathways.

The primary objective of this study was to investigate the key mechanisms by which ALBC regulates the activation of the NLRP3 inflammasome and influences macrophage M1/M2 phenotype polarization to elucidate further its molecular regulatory pathways in promoting wound healing in DFUs. By establishing a HG cell model, a murine wound model, and collecting clinical DFU samples, we systematically validated the immunomodulatory potential of ALBC. This study aimed to reveal the immunological properties of this novel material, thereby providing new theoretical support and molecular targets for individualized DFU treatment strategies while promoting the transformation of ALBC from an “antimicrobial material” to an “immunoregenerative material.” The ultimate goal was to offer a more effective, safer, and widely applicable therapeutic approach for DFUs and other chronic non‐healing wounds, thereby improving clinical outcomes and the quality of life for patients.

## 2 MATERIALS AND METHODS

### 2.1 Mouse husbandry

db/db diabetic gene‐deficient mice (NM‐KI‐00001) were purchased from Shanghai Southern Model Organisms Biotechnology Co., Ltd. Both male and female mice were used in equal numbers and treated at 7–9 weeks of age. Prior to the experiment, the mice were acclimated for 1 week and housed under controlled conditions (22°C, 60% humidity, 12‐h light/dark cycle). All mice were fasted for 3–4 h before the experiments and weighed between 30 and 35 g. Each group included eight mice, which were randomly assigned, with littermates used as controls. All animal studies were conducted in accordance with the protocol approved by the Animal Ethics Committee of Zibo Central Hospital (No. 2024 Research No. 069). All procedures followed the international guidelines for the ethical use of laboratory animals, complied with national regulations, and adhered to the ARRIVE guidelines.

### 2.2 Mouse modeling and treatment

Gentamicin (60214ES25, Yeasen Biotechnology) and vancomycin (60213ES96, Yeasen Biotechnology) were mixed with 40 g of PMMA bone cement powder (1200/I, Tecres), followed by manual mixing with the cement liquid. The high‐dose ALBC (ALBC‐H) contained 500 mg gentamicin and 4 g vancomycin; the medium‐dose ALBC (ALBC‐M) contained 500 mg gentamicin and 2 g vancomycin; the low‐dose ALBC (ALBC‐L) contained 500 mg gentamicin and 1 g vancomycin.

After 1 week of acclimatization, full‐thickness skin defect models were established in mice. Briefly, mice were anesthetized with 1% pentobarbital, the dorsal hair was shaved, and a circular incision with a diameter of 0.8 cm was made at the center of the back. Each mouse was housed individually, and the wound area was topically treated with ALBC‐L, ALBC‐M, or ALBC‐H. On Day 7, the bone cement blocks were removed, and the wound was flushed with sterile saline to ensure no residue remained. Sterile gauze was used to cover the wound, and photographs were taken on Days 0, 7, and 14 to monitor healing progression. On Day 14, full‐thickness skin tissues surrounding the wound edges were collected and stored at −80°C for subsequent experiments.^29^, ^30^ Immediately after treatment with ALBC‐H, some mice received multiple‐point injections of the NLRP3 activator Nigericin (4 mg/kg, HY‐100381, MCE)^31^ or the NLRP3 inhibitor MCC950 (20 mg/kg, HY‐12815, MCE)^32^ around the wound area. The same dose was administered once daily for five consecutive days. Wound images were captured using a digital camera on Days 7 and 14 to record the healing process. Wound area was measured using ImageJ software, and the wound closure percentage was calculated using the following formula: *P*(%) = [(A0 − A1)/A0] × 100, where *P* is the percentage of wound closure, and A0 and A1 represent the initial and residual wound areas, respectively.^33^ On Day 14, full‐thickness skin tissues surrounding the wound margins were harvested and stored at −80°C for further analysis.

Grouping of mice (*n* = 8): Model group (mice with skin defect model), ALBC‐L group (mice with skin defect treated with ALBC‐L), ALBC‐M group (mice with skin defect treated with ALBC‐M), ALBC‐H group (mice with skin defect treated with ALBC‐H), and ALBC‐H + Nigericin group (mice with skin defect treated with ALBC‐H and additionally administered the NLRP3 activator Nigericin).

### 2.3 Cell treatment

RAW264.7 cells (CL‐0190) were purchased from Wuhan Procell Life Science & Technology Co., Ltd. The selected cell line was cultured in Dulbecco's Modified Eagle Medium (PM150210, Procell) supplemented with 10% (v/v) fetal bovine serum (164210, Procell) and 1% (v/v) penicillin–streptomycin (PB180120, Procell). Cells were maintained under standard conditions (37°C, 5% CO_2_). When cell confluence reached approximately 80%, the cells were passaged using 0.25% trypsin, and passages 2–7 were used for subsequent experiments. The control group cells were cultured in 5.5 mmol/L glucose medium. For the HG group, D‐glucose (ST1228, Beyotime) was added to the standard culture medium to reach a final concentration of 30 mmol/L, mimicking the diabetic HG environment. NLRP3 inflammasome activation was successfully induced by pretreating RAW264.7 macrophages with 1 μg/mL lipopolysaccharide (LPS) for 4 h, followed by stimulation with 5 mM adenosine triphosphate (ATP) for 20 min.

#### 2.3.1 ALBC treatment

When RAW264.7 cells adhered to the culture plate surface, the culture medium was replaced with an ALBC extract‐containing medium. Preparation of the extract medium involved immersing ALBC‐L, ALBC‐M, and ALBC‐H in HG cell culture medium at a weight‐to‐volume ratio of 0.1 mg/mL. The mixture was shaken continuously at 37°C for 24 h to obtain the respective ALBC extract media. The extracts were centrifuged at 1000 rpm for 5 min and filtered through a 0.2‐μm pore membrane.

Cells were divided into the following experimental groups: Control, HG, HG + ALBC‐L, HG + ALBC‐M, and HG + ALBC‐H.

### 2.4 Histological analysis

Collected wound edge tissues were fixed in 4.0% paraformaldehyde solution at room temperature, embedded in paraffin, and sectioned into 4‐μm‐thick slices. The tissue sections were heated at 60°C for 1 h, followed by deparaffinization and rehydration through a series of xylene and graded ethanol immersions. Hematoxylin and eosin (H&E) staining was subsequently performed at room temperature (hematoxylin for 5 min; eosin for 2 min). An optical microscope (magnification ×200) was used to evaluate inflammatory cell infiltration, neoepidermis formation, and neovascularization in the wound areas.

### 2.5 Immunohistochemistry

Fixed mouse wound tissues were processed into paraffin sections, placed on glass slides, and melted on a slide warmer at 60°C. The sections were then baked and deparaffinized in xylene jars, followed by rehydration. Antigen retrieval was performed by steaming the sections in 0.01 mol/L citrate‐sodium citrate buffer for 30 min. Endogenous peroxidase activity was blocked using 3% hydrogen peroxide. Primary antibodies against NLRP3 (CSB‐PA209407, 1:100, Cusabio) and Caspase‐1 (CSB‐PA001227, 1:100, Cusabio) were diluted according to the manufacturer's instructions, applied to the slides, and incubated overnight at 4°C. The horseradish peroxidase (HRP)‐conjugated goat anti‐rabbit IgG (H + L) secondary antibody (ab6721, 1:1000, Abcam) was diluted in PBS at 1:50 and incubated with the slides at 37°C for 30 min. TMB chromogenic solution was added and incubated in the dark for 60 min. Finally, images were captured using a fluorescence microscope (magnification 20 × 10), and Image‐Pro Plus 6.0 software was used to analyze the data and calculate the percentage of positively stained cells.

### 2.6 Enzyme‐linked immunosorbent assay

Following the manufacturer's instructions, enzyme‐linked immunosorbent assay (ELISA) kits (Merck, Shanghai, China) were used to measure cytokine levels in mouse serum and cell supernatants at 48 h. The following detection kits were used: IL‐1β (BMS6002‐2, Invitrogen), IL‐4 (ab100710, Abcam), IL‐6 (ab100713, Abcam), IL‐10 (ab46103, Abcam), and IL‐18 (PI553, Beyotime). Relative absorbance was measured at 450 nm using a BioTek Synergy HTX microplate reader (BioTek, Vermont, USA).

### 2.7 Immunofluorescence assay

Paraffin‐embedded tissue sections were deparaffinized and subjected to antigen retrieval by heating in citrate buffer (Beyotime Biotechnology, Haimen, China) at 95°C in a water bath for 15 min, then cooling to room temperature. The sections were washed thrice with PBS (pH 7.4) for 5 min each. After blocking with blocking buffer at room temperature for 2 h, the sections were incubated overnight at 4°C with primary antibodies against TNF‐α (ab1793, 5 μg/mL, Abcam), iNOS (ab283655, 1:50, Abcam), CD206 (sc‐58986, 1:500, Santa Cruz), and CD163 (ab316218, 1:50, Abcam). After washing with PBS three times for 5 min, the sections were incubated for 50 min at room temperature in the dark with Alexa Fluor 488‐conjugated goat anti‐mouse IgG (ab150113, 1:200, Abcam) and Alexa Fluor 647‐conjugated donkey anti‐rabbit IgG H&L (ab150075, 1:200, Abcam) secondary antibodies. The sections were washed and incubated with DAPI (ab104139, Abcam) for 10 min. After washing, the autofluorescence quenching reagent (G1221‐5ML, Servicebio) was applied for 5 min and then rinsed under running water for 10 min. Finally, sections were mounted using an anti‐fade mounting medium (G1401‐25ML, Servicebio). Images were captured using a fluorescence microscope (Leica Microsystems, Germany) at 400× magnification. For each section, three to five fields of view were selected. The number of positive cells in each field was analyzed using ImageJ software, and the average number of positive cells per section was calculated.

### 2.8 Flow cytometry

Tissues were collected into tissue culture dishes containing 10 mL of flow cytometry staining buffer. A 3 mL syringe was mechanically used to dissociate the tissues into single‐cell suspensions. A cell strainer was placed on top of a 15 mL conical tube to filter the suspension and remove cell clumps and debris. The filtered cell suspension was centrifuged at 300–400 × g for 5 min at 2–8°C. The supernatant was discarded, and the cell pellet was resuspended in an appropriate volume of staining buffer. To block nonspecific Fc receptor‐mediated interactions, 20 μL of purified Fc receptor binding inhibitor was added to every 100 μL of single‐cell suspension and incubated at 2–8°C or room temperature for 10–20 min. The staining antibody mixture was prepared in flow cytometry staining buffer to a final volume of 50 μL and added to the cells, followed by gentle vortexing. The mixture was incubated at room temperature for 20–30 min in the dark. The antibodies used included TNF‐α (ab1793, 1:10, Abcam), iNOS (ab283655, 1:600, Abcam), CD206 (sc‐58986, 1:500, Santa Cruz), and CD163 (ab316218, 1:500, Abcam). After staining, the cells were washed twice and analyzed using a flow cytometer. Data were analyzed using FlowJo software.

### 2.9 Reverse transcription quantitative PCR

Macrophages from each group were collected, and total RNA was extracted using Trizol reagent (15596018CN, Invitrogen, USA). To synthesize cDNA, reverse transcription was performed using the PrimeScript™ RT reagent kit (RR047Q, Takara, Japan). Reverse transcription quantitative PCR (RT‐qPCR) was conducted using the BeyoFast™ SYBR Green One‐Step qRT‐PCR kit (D7268S, Beyotime) on an ABI PRISM 7500 Real‐Time PCR system (Applied Biosystems, Thermo Fisher), with all reactions performed in triplicate. Relative mRNA expression was calculated using the 2^−ΔΔCt^ method, where ΔΔCt = (Ct_target gene in experimental group − Ct_reference gene in experimental group) − (Ct_target gene in control group − Ct_reference gene in control group). GAPDH was used as the internal reference gene. RT‐qPCR reactions were carried out on the StepOnePlus system (Applied Biosystems, California, USA) with the following cycling conditions: an initial cycle at 95°C for 15 min, followed by 40 cycles of 95°C for 10 s and 60°C for 60 s. Primer sequences are listed in Table S1. All reagents and materials used in the above experiments were purchased from Wuhan Servicebio Technology Co., Ltd.

### 2.10 Western blot

Tissues and cells were lysed using RIPA lysis buffer (P0013B, Beyotime, China) to extract total protein. Protein concentrations were quantified using a BCA protein assay kit (23227, Thermo Fisher Scientific, USA). Equal amounts of protein samples were separated by SDS‐PAGE and transferred onto PVDF membranes (IPVH00010, Millipore, USA). Membranes were blocked with 5% non‐fat milk for 2 h and washed three times with TBST containing Tween 20 (10 min per wash). The membranes were then incubated overnight at 4°C with primary antibodies (detailed antibody information is provided in Table S2). After washing, the membranes were incubated with HRP‐conjugated goat anti‐rabbit secondary antibodies (ab6721, 1:2000, Abcam, UK) at room temperature for 2 h and visualized using an enhanced chemiluminescence detection system (iBright FL1500; Thermo Fisher). GAPDH was used as the internal control. Each experiment was repeated three times. Relative protein levels were calculated as the ratio of the grayscale intensity of the target protein band to that of the GAPDH band.

### 2.11 Statistical analysis

All data were analyzed using Prism 9.5.0 statistical software. Quantitative data were expressed as mean ± standard deviation. Comparisons between two groups were performed using independent sample *t*‐tests, while comparisons among multiple groups were evaluated by one‐way analysis of variance (ANOVA). Categorical data were expressed as counts or percentages and analyzed using the chi‐square test. A *p*‐value <0.05 was considered statistically significant.

## 3 RESULTS

### 3.1 ALBC accelerated wound healing in diabetic mice

**FIGURE 1:**
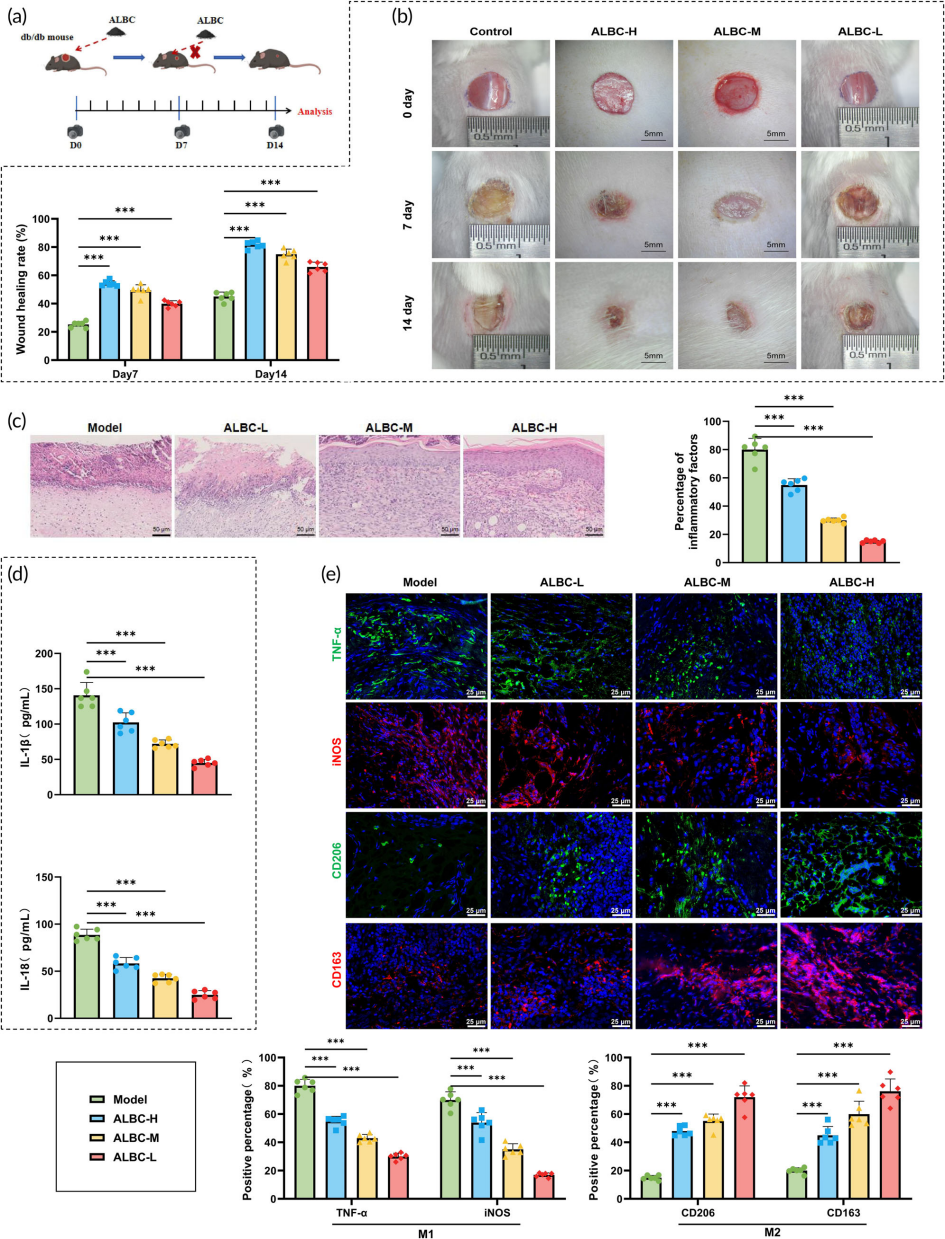
Effects of antibiotic‐loaded bone cement (ALBC) on wound healing in diabetic mice. (a) Schematic diagram of the experimental protocol for ALBC treatment in the diabetic mouse skin defect model, created by Biorender; (b) wound healing status in each group on Days 7 and 14; (c) hematoxylin and eosin staining of skin tissue sections in each group; (d) serum levels of IL‐1β and IL‐18 in each group; (e) immunofluorescence detection of TNF‐α, iNOS, CD206, and CD163 expression in mouse skin tissues. *N* = 6; *** indicates *p <* 0.001 between groups. ALBC‐H, high‐dose ALBC; ALBC‐L, low‐dose ALBC; ALBC‐M, medium‐dose ALBC.

To investigate the molecular mechanism of ALBC in the treatment of DFUs, a circular full‐thickness skin wound with a diameter of 0.8 cm was created on the dorsal skin of db/db diabetic gene‐deficient mice. ALBC‐H, ALBC‐M, and ALBC‐L were applied locally to the wound sites. On Day 7, the bone cement blocks were removed, and wound healing was monitored until Day 14 (Figure 1a). Throughout the healing process, the progression of wound closure was documented using digital photography. The results showed that, compared with the model group, mice treated with ALBC exhibited significantly higher wound healing rates on both Day 7 and Day 14. Moreover, the healing rate increased in a dose‐dependent manner, with higher doses of ALBC leading to faster wound closure (Figure 1b, Table 1). These findings indicated that ALBC accelerated wound healing in DFUs and demonstrated a clear dose‐dependent effect. H&E staining and histological analysis revealed severe inflammatory cell infiltration and incomplete tissue repair in the model group. In contrast, the ALBC‐treated groups—particularly the ALBC‐H group—showed a marked reduction in inflammatory cell infiltration and improved neoepidermis formation and neovascularization (Figure 1c). These histological changes further supported the effectiveness of ALBC in promoting wound healing.

**TABLE 1:** Effect of antibiotic bone cement on wound healing rate of diabetic foot mice.

Group	Day 7 wound reduction (%)	Day 14 wound healing (%)
	(mean ± SD)	(mean ± SD)
Model	25.78 ± 2.42	46.00 ± 2.26
ALBC‐H	53.78 ± 2.61	81.22 ± 3.07
ALBC‐L	39.06 ± 3.41	62.39 ± 2.55
ALBC‐M	48.96 ± 2.03	74.88 ± 3.36

Abbreviations: ALBC, antibiotic‐loaded bone cement; ALBC‐H, high‐dose ALBC; ALBC‐L, low‐dose ALBC; ALBC‐M, medium‐dose ALBC.

We measured the levels of inflammatory cytokines in serum using ELISA. The results showed that, compared with the model group, mice treated with ALBC exhibited significantly reduced levels of pro‐inflammatory cytokines IL‐1β and IL‐18, with the most pronounced reduction observed in the ALBC‐H group (Figure 1d). Macrophages are key immune cells, and their polarization toward the anti‐inflammatory M2 phenotype benefits wound healing.^34^ Immunofluorescence (IF) analysis revealed that ALBC treatment markedly influenced macrophage M1/M2 phenotype polarization compared with the model group. The expression of M1 markers TNF‐α and iNOS was significantly downregulated, while M2 markers CD206 and CD163 were significantly upregulated. Notably, the M1‐to‐M2 shift was most evident in the ALBC‐H group (Figure 1e).

These results indicated that ALBC promoted wound healing in diabetic mice by modulating macrophage M1/M2 polarization and attenuating the inflammatory response.

### 3.2 ALBC regulated macrophage polarization and inflammation levels in RAW 264.7 cells in vitro

**FIGURE 2:**
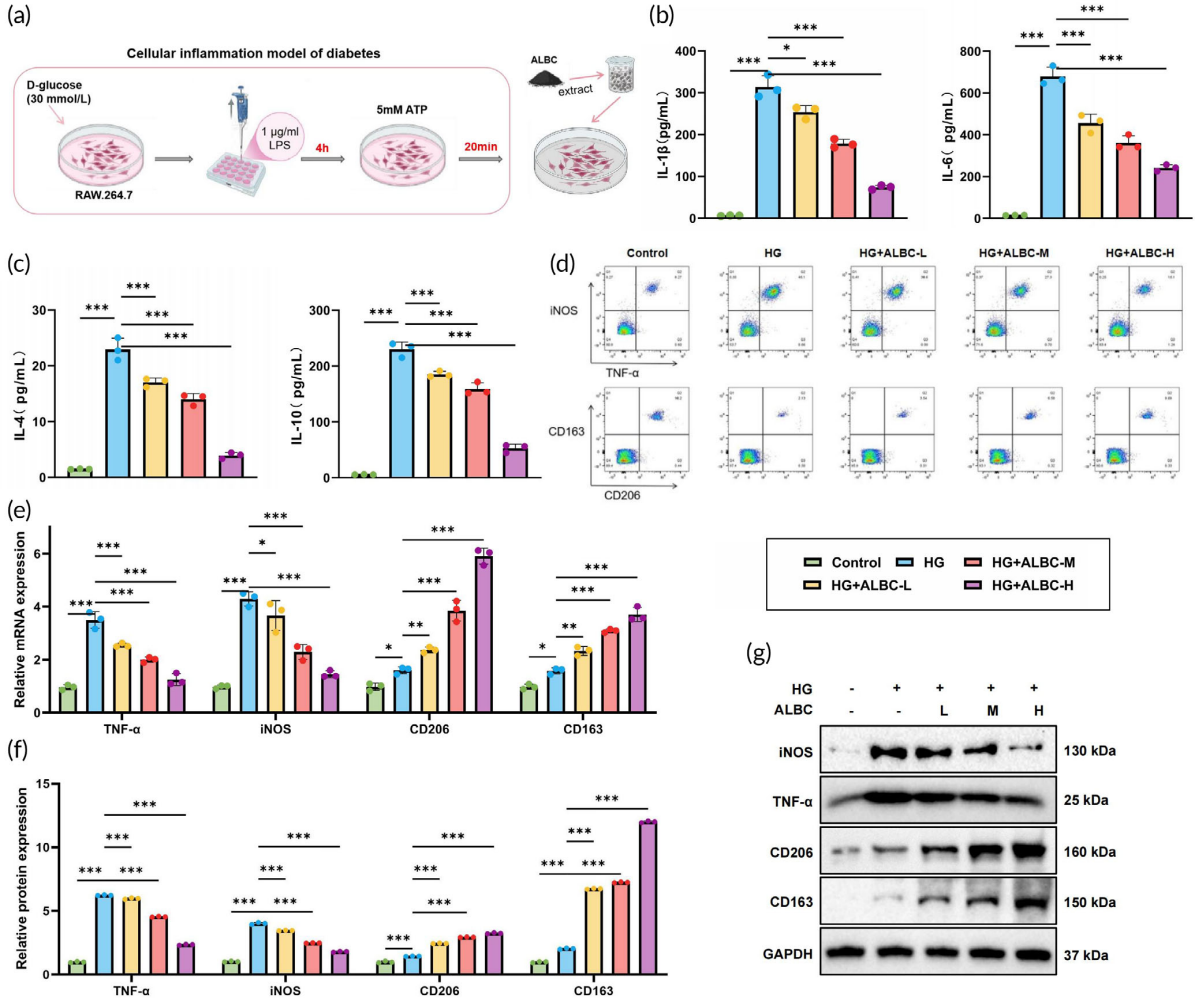
Effects of antibiotic‐loaded bone cement (ALBC) on RAW 264.7 cell polarization and inflammation. (a) Schematic diagram of the experimental protocol for ALBC treatment in a diabetic foot ulcer RAW 264.7 inflammatory model, created by Biorender; (b) levels of pro‐inflammatory cytokines IL‐1β and IL‐6 in the cell supernatant; (c) levels of anti‐inflammatory cytokines IL‐4 and IL‐10; (d) flow cytometric analysis of macrophage phenotype markers (TNF‐α, iNOS, CD206, and CD163) (*n* = 3); (e) Reverse transcription quantitative PCR analysis of TNF‐α, iNOS, CD206, and CD163 expression; (f, g) western blot analysis of TNF‐α, iNOS, CD206, and CD163 protein levels. Cell experiments were repeated three times. *p < 0.05, **p < 0.01, ***p < 0.001 between groups. HG, high‐glucose.

We simulated the physiological conditions of DFUs in vitro by mimicking the HG diabetic environment. RAW 264.7 macrophages were pretreated with LPS for 4 h and subsequently stimulated with ATP for 20 min to establish a DFU‐associated inflammatory cell model (HG model). ALBC‐H, ALBC‐M, and ALBC‐L extracts were then applied to the RAW 264.7 cells to evaluate the biological effects of ALBC on macrophages (Figure 2a). ELISA results showed that compared with the control group, the HG group exhibited significantly increased levels of pro‐inflammatory cytokines IL‐1β and IL‐6 and decreased levels of anti‐inflammatory cytokines IL‐4 and IL‐10 in the cell supernatants. Treatment with ALBC significantly suppressed the upregulation of pro‐inflammatory cytokines and reversed the downregulation of anti‐inflammatory cytokines in a dose‐dependent manner, with the most pronounced effect observed in the ALBC‐H group (Figure 2b,c).

We further assessed macrophage phenotype polarization. Flow cytometry results revealed that, compared with the control group, the proportion of M1 macrophages (TNF‐α^+^/iNOS^+^) in HG‐treated RAW 264.7 cells was significantly increased, while the proportion of M2 macrophages (CD206^+^/CD163^+^) was significantly decreased. ALBC treatment effectively inhibited the increase in M1 macrophages and promoted the recovery of M2 macrophages, with the ALBC‐H group showing the most prominent effect (Figure 2d). In addition, RT‐qPCR and Western blot analyses were performed to evaluate the expression of M1 markers (TNF‐α and iNOS) and M2 markers (CD206 and CD163) at both gene and protein levels. The results were consistent with the trends observed by flow cytometry (Figure 2e–g).

These findings demonstrated that ALBC effectively promoted macrophage polarization from the pro‐inflammatory M1 phenotype to the anti‐inflammatory M2 phenotype.

### 3.3 ALBC inhibited NLRP3 inflammasome activation

**FIGURE 3:**
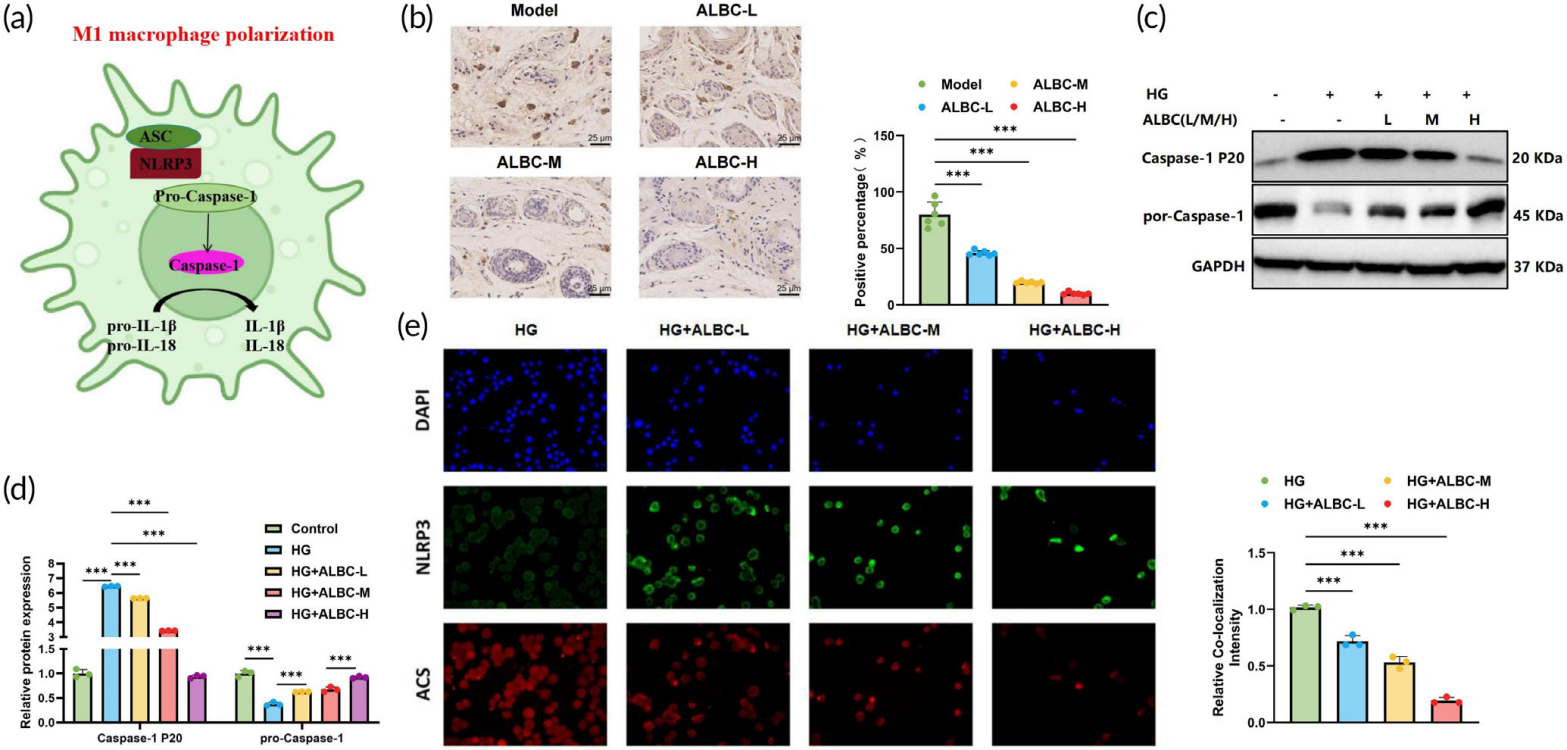
Regulation of NLRP3 inflammasome activation by antibiotic‐loaded bone cement (ALBC). (a) Schematic diagram of NLRP3 inflammasome activation; (b) immunohistochemistry detection of NLRP3 and Caspase‐1 expression in full‐thickness skin tissue surrounding the wound edge in mice; (c, d) western blot analysis of Pro‐Caspase‐1 and cleaved Caspase‐1 expression in cells; (e) IF detection of NLRP3 and apoptosis‐associated speck‐like protein containing a CARD (ASC) co‐localization. *N* = 6; cell experiments were repeated three times; ***p < 0.001 between groups. ALBC‐H, high‐dose ALBC; ALBC‐L, low‐dose ALBC; ALBC‐M, medium‐dose ALBC; HG, high‐glucose.

The NLRP3 inflammasome functions as a critical molecular switch in regulating macrophage polarization. Its overactivation promotes polarization toward the pro‐inflammatory M1 phenotype while suppressing M2 phenotype polarization, thereby exacerbating inflammation (Figure 3a). In DFUs, NLRP3‐driven M1 polarization can delay wound healing. We examined the effect of ALBC on NLRP3 inflammasome activation in both in vivo and in vitro DFU models.

In the DFU mouse model, immunohistochemistry (IHC) analysis revealed that, compared with the model group, ALBC treatment significantly reduced NLRP3 expression levels in full‐thickness skin tissues around the wound edges, with the most notable reduction observed in the ALBC‐H group (Figure 3b). In the DFU RAW 264.7 cell model, Western blot analysis (Figure 3c,d) showed that, compared with the control group, Pro‐Caspase‐1 levels decreased in the HG group, while levels of cleaved Caspase‐1 fragments increased, indicating activation of Caspase‐1. Following ALBC treatment, Caspase‐1 activation was markedly suppressed, and the expression of cleaved Caspase‐1 fragments was nearly eliminated in the ALBC‐H group. Furthermore, IF results showed strong co‐localization signals of NLRP3 and ASC in the HG group. In contrast, these signals were significantly reduced in the ALBC‐treated groups, particularly in the ALBC‐H group, indicating strong inhibition of NLRP3 inflammasome assembly (Figure 3e). These results demonstrated that ALBC effectively suppressed the activation of the NLRP3 inflammasome.

### 3.4 ALBC inhibited NLRP3 inflammasome activation to treat DFUs in mice

**FIGURE 4:**
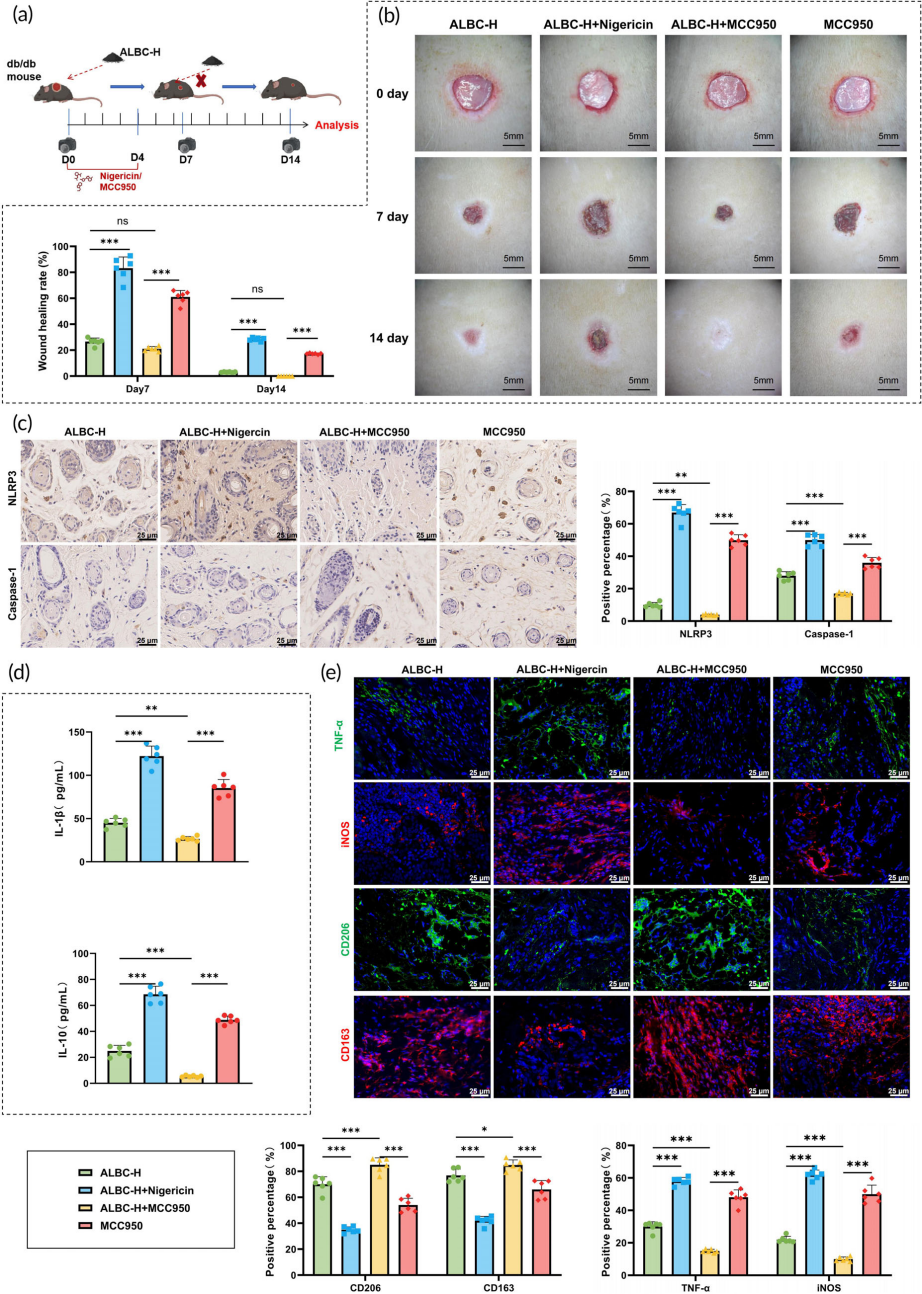
Role of the NLRP3 inflammasome in the therapeutic effect of antibiotic‐loaded bone cement (ALBC) in diabetic foot ulcer (DFU) mice. (a) Schematic diagram of the treatment protocol in which diabetic foot ulcer (DFU0 mice received high‐dose antibiotic‐loaded bone cement [ALBC‐H] in combination with the NLRP3 activator Nigericin or the inhibitor MCC950); (b) wound healing rates on Days 7 and 14 in each group; (c) immunohistochemistry analysis of NLRP3 and Caspase‐1 expression in skin tissues from each group; (d) enzyme‐linked immunosorbent assay analysis of IL‐1β and IL‐18 cytokine levels; (e) immunofluorescence detection of TNF‐α, iNOS, CD206, and CD163 protein expression in mouse skin tissues. *N* = 6; *p < 0.05, **p < 0.01, ***p < 0.001 between groups.

We further investigated the role of the NLRP3 inflammasome in the therapeutic effect of ALBC by treating DFU mice with the NLRP3 activator Nigericin or inhibitor MCC950 in combination with ALBC‐H (Figure 4a). During the wound healing process, mice in the ALBC‐H + Nigericin group showed a significantly reduced wound healing rate compared with the ALBC‐H group, whereas treatment with MCC950 further improved wound healing (Figure 4b). IHC results revealed that, compared with the ALBC‐H group, Nigericin treatment significantly increased NLRP3 and Caspase‐1 expression levels in skin tissues, while MCC950 treatment led to a marked decrease in their expression (Figure 4c). ELISA results showed that Nigericin significantly elevated serum IL‐1β and IL‐18 levels, whereas MCC950 further improved the inflammatory profile observed in the ALBC‐H group (Figure 4d). Additionally, IF analysis demonstrated that, compared with the ALBC‐H group, the ALBC‐H + Nigericin group exhibited significantly increased expression of M1 markers TNF‐α and iNOS, and decreased expression of M2 markers CD206 and CD163. Conversely, in the ALBC‐H + MCC950 group, TNF‐α and iNOS expression was significantly reduced, while CD206 and CD163 levels were markedly elevated (Figure 4e).

These results indicated that the NLRP3 activator promoted NLRP3 inflammasome activation, enhanced M1 macrophage polarization, and exacerbated inflammation, thereby reversing the beneficial effects of ALBC on wound healing in DFU mice. In contrast, co‐treatment with the NLRP3 inhibitor MCC950 and ALBC further accelerated wound healing in DFU mice.

### 3.5 Clinical validation of ALBC in DFU patient samples

**FIGURE 5:**
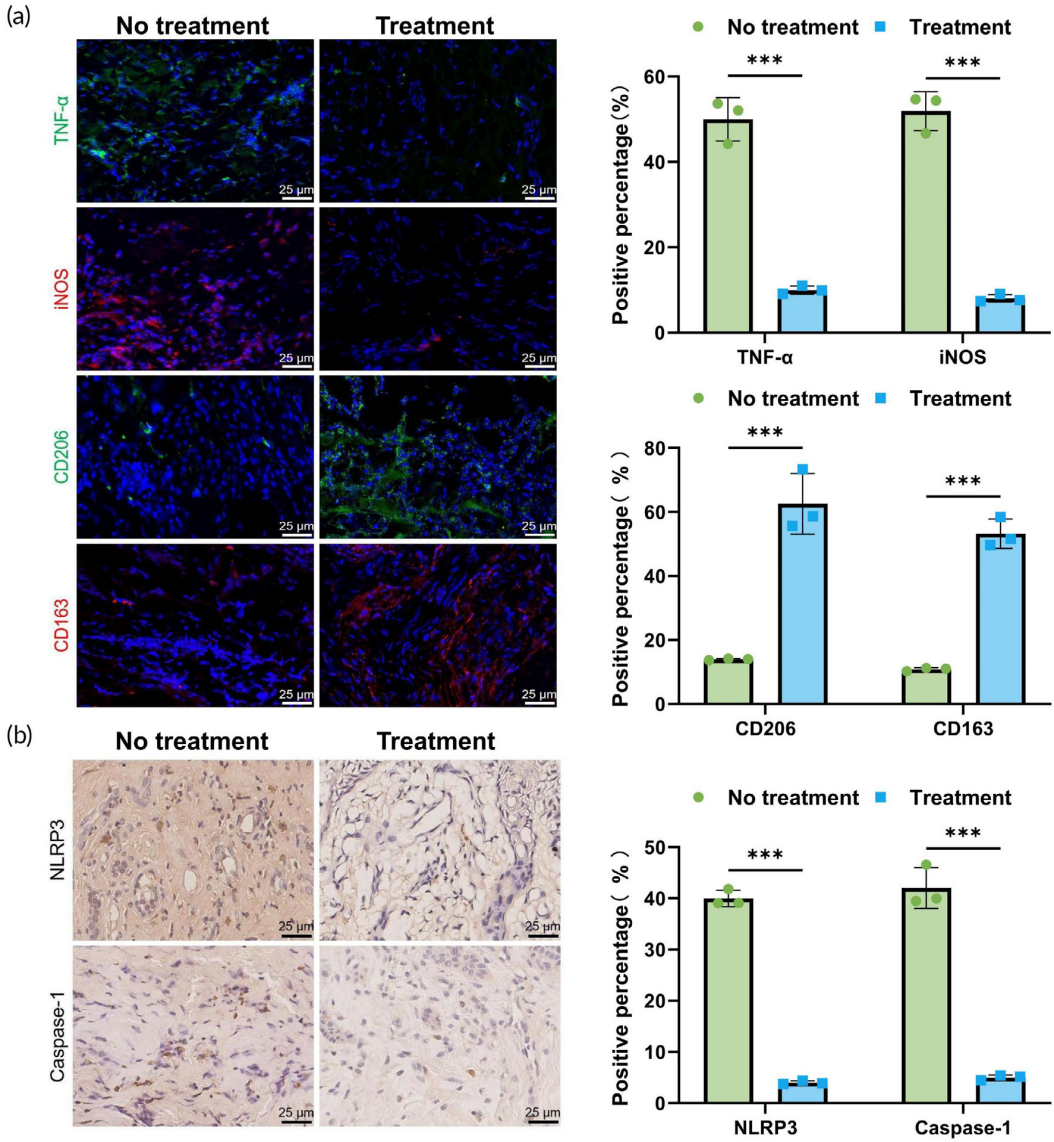
Effects of antibiotic‐loaded bone cement on wound tissues of diabetic foot ulcer (DFU) patients. (a) Immunofluorescence detection of M1 and M2 macrophage phenotype markers in DFU patient wound tissues; (b) immunohistochemistry analysis of NLRP3 and Caspase‐1 expression in DFU patient wound tissues. *N* = 3; ***p < 0.001 between groups.

We next validated the potential clinical efficacy of ALBC in tissue samples from patients with DFUs. Wound tissue samples were collected from DFU patients before and after ALBC treatment. The expression of M1 and M2 macrophage phenotype markers was assessed using IF staining. The results showed that, compared with patients who did not receive ALBC treatment, those treated with ALBC exhibited a significant decrease in M1 marker expression and a marked increase in M2 marker expression (Figure 5a). These findings indicated that ALBC effectively promoted macrophage polarization toward the M2 phenotype in clinical settings, thereby reducing local inflammation and facilitating wound healing.

To further investigate the regulatory effect of ALBC on the NLRP3 inflammasome, we examined the expression of NLRP3 and Caspase‐1 in DFU wound tissues using IHC. The results showed that, in samples from patients who had not received ALBC treatment, the expression levels of NLRP3 and Caspase‐1 were significantly elevated. In contrast, patients who received ALBC treatment exhibited a marked reduction in the expression of both proteins, particularly in the ALBC‐H group (Figure 5b). These findings further confirmed that ALBC mitigated the inflammatory response in DFU tissues by inhibiting the activation of the NLRP3 inflammasome.

Clinical data analysis showed a significant correlation between NLRP3 inflammasome expression and macrophage M1/M2 phenotype polarization. High NLRP3 expression positively correlated with M1 markers TNF‐α and iNOS expression and negatively correlated with M2 markers CD206 and CD163 (Table 2). These results further support the role of ALBC in modulating the NLRP3 inflammasome, promoting macrophage polarization, and enhancing wound healing in DFU.

**TABLE 2:** Correlation analysis of NLRP3 inflammasome expression and M1/M2 marker expression in macrophages.

Comparison	Correlation coefficient (*r*)	*p*‐Value
NLRP3 versus TNF‐β	0.72	<0.05
NLRP3 versus iNOS	0.7	<0.05
NLRP3 versus CD206	−0.68	<0.05
NLRP3 versus CD163	−0.69	<0.05

*Note*: The data were expressed as correlation coefficient *r*, and *p* < 0.05 indicated that the correlation was statistically significant.

In summary, the results demonstrated that ALBC significantly promoted wound healing and reduced local inflammation in DFU patients by regulating NLRP3 inflammasome activation and macrophage M1/M2 polarization, indicating its promising potential for clinical application.

## 4 DISCUSSION

ALBC, as a local controlled‐release system, was initially used in orthopedic surgery to prevent and treat postoperative infections. Its core advantage lies in achieving high local antibiotic concentrations while avoiding systemic toxicity.^35^ In recent years, several studies have attempted to expand its applications to chronic wounds and soft tissue infections,^36^ but most have focused on its antimicrobial activity and physical barrier function,^37^ with limited exploration of its potential role in modulating immune and inflammatory responses.^38^ This study was the first to introduce ALBC into the field of DFU wound repair systematically and investigated its roles in regulating immune cell function, suppressing key inflammatory pathways, and promoting tissue regeneration. We proposed and validated a novel therapeutic mechanism—the ALBC‐NLRP3 inflammasome‐macrophage polarization axis. This mechanism broadens the scope of ALBC applications and provides theoretical and experimental support for its use in treating chronic, non‐healing wounds.

The NLRP3 inflammasome, an essential intracellular inflammatory signaling platform, has been repeatedly shown to be closely associated with impaired wound healing in DFU and other chronic inflammatory diseases.^21^ Previous studies have demonstrated that HG environments can induce ROS production and mitochondrial damage,^39^ activate NLRP3, and upregulate Caspase‐1 activity,^40^ ultimately promoting the maturation and release of pro‐inflammatory cytokines such as IL‐1β and IL‐18, leading to sustained inflammation.^41^ Our study confirmed that ALBC significantly suppressed the expression of NLRP3 and Caspase‐1 in DFU mouse models and further clarified the critical role of NLRP3 in ALBC‐mediated wound healing through intervention experiments using Nigericin and MCC950. We observed consistent trends in clinical patient tissue samples, enhancing our findings' clinical relevance and credibility. This closed‐loop design, spanning from mechanistic validation to clinical observation, represents a key strength of this study and offers a novel perspective for material‐based modulation of the NLRP3 inflammasome.

In terms of macrophage functional regulation, this study also yielded significant findings. The direction of macrophage polarization influences local inflammation levels and plays a central role in regulating tissue regeneration and angiogenesis.^17^ Previous studies have shown that the HG diabetic environment induces sustained activation of M1 macrophages, which express high levels of pro‐inflammatory factors such as TNF‐α and iNOS, thereby hindering the replacement by M2 macrophages and the activation of wound healing signals.^42^, ^43^, ^44^ In our study, we observed in animal models, RAW264.7 cell inflammation models, and clinical patient samples that ALBC significantly downregulated M1 markers while upregulating M2 markers such as CD206 and CD163. More importantly, this shift was accompanied by increased expression of anti‐inflammatory cytokines IL‐10 and IL‐4 and a concurrent decrease in pro‐inflammatory factors, suggesting that ALBC may function not only as a passive drug delivery platform but also actively modulate macrophage function through its material properties or bioactive components in the extract. This discovery provides new evidence supporting the development of immune‐regulatory biomaterials and offers a theoretical basis for future material‐mediated immunoregenerative therapies.

It should be noted that DFUs are characterized by a chronic non‐healing state, directly linked to functional alterations in immune responses. In addition to the macrophages highlighted in this study, mast cells also play a pivotal role, with intricate interactions reported between the two cell types.^45^ Previous studies have shown that inhibition of mast cell degranulation promotes macrophage polarization toward the reparative M2 phenotype, thereby enhancing angiogenesis and collagen deposition.^46^, ^47^, ^48^ Conversely, M1 macrophages may activate quiescent mast cells to release pro‐inflammatory mediators, perpetuating a feedback loop of sustained inflammation and impaired wound repair.^45^ Furthermore, evidence suggests that alterations in key genes may jointly regulate mast cell and macrophage infiltration and polarization patterns.^49^ These findings indicate that mast cell–macrophage crosstalk exerts a critical influence on the immune microenvironment of DFUs. Future studies should investigate the impact of ALBC on overall immune infiltration, aiming to elucidate the functional interactions between mast cells, macrophages, and other immune cell subsets, thereby enriching the mechanistic basis of its therapeutic effects.

In addition, we observed that ALBC positively influenced tissue structural remodeling. H&E staining showed that in the ALBC‐H treatment group, inflammatory cell infiltration was markedly reduced, accompanied by more pronounced granulation tissue formation, increased neovascularization, and improved epithelial regeneration. These results suggested that, beyond suppressing inflammation, ALBC may also indirectly promote tissue regeneration. For example, M2 macrophages are known to secrete factors such as TGF‐β and VEGF to promote angiogenesis and basement membrane formation; ALBC may act through this pathway by directing macrophage polarization. Although this study did not further quantify the expression changes of these repair‐associated factors following ALBC treatment, this represents a promising direction for future research.

It is worth emphasizing that the design of this study integrated data from animal models, cell models, and clinical samples, providing a high level of evidence and strong translational relevance. In the DFU mouse model, we quantified wound healing rates and dynamically tracked immunological changes throughout the wound repair process. In the RAW264.7 cell experiments, we systematically analyzed macrophage polarization using flow cytometry, qPCR, and Western blot. In clinical samples, key protein expression trends were validated using IF and IHC, and their correlations with healing indicators were further analyzed using statistical methods. This multi‐tiered, multi‐method experimental design significantly enhanced the reliability of our findings and provided robust data support for the future clinical translation of ALBC.

Compared with existing studies, this research offered multiple innovative and groundbreaking contributions. First, ALBC was not merely positioned as an antimicrobial material but redefined as an immunomodulatory material, representing a major conceptual extension of its functional role. Second, this study was the first to jointly investigate the NLRP3 inflammasome and macrophage polarization as a central axis, revealing their interactive roles in the chronic inflammatory microenvironment of DFUs and validating the axis as both a functional and therapeutically targetable pathway. Third, by incorporating a dose gradient and functional control groups, we preliminarily proposed an optimal immunoregulatory dosage range for ALBC, providing a reference for future clinical dose–response applications. Finally, integrating basic experimental data with clinical tissue samples greatly enhanced the study's conclusions' extrapolative value and real‐world applicability.

Although this study systematically investigated the role of ALBC in regulating NLRP3 inflammasome activation and macrophage polarization and validated its mechanism in promoting wound healing in DFUs, certain limitations remained. First, the ALBC used in this study was a conventional formulation with complex release components. The specific bioactive constituents responsible for immune modulation have not yet been identified, and no isolation or functional characterization of the active components has been performed. Second, although the results from animal and cell models were largely consistent with those from clinical samples, the clinical sample size was relatively small, and the study lacked prospective clinical trial validation. Therefore, the findings did not fully evaluate the broad applicability and inter‐individual variability across diverse patient populations. Third, the observation window was limited to 14 days, reflecting only the early to mid‐stages of wound healing, without addressing long‐term healing quality, biocompatibility, or potential chronic inflammation caused by material degradation. Furthermore, this study primarily focused on macrophages and the NLRP3 pathway, whereas the immune microenvironment of DFU wounds involves various immune cells and complex inflammatory networks. Other potentially involved pathways, such as NF‐κB, STAT6, and TLRs, were not explored in depth, limiting the comprehensiveness of mechanistic interpretation.

Based on the findings of this study, future research can be expanded along several dimensions. On one hand, key immunomodulatory components within ALBC should be further isolated and identified, and their mechanisms of action and molecular targets clarified to provide a theoretical basis for subsequent material modification and precision drug design. On the other hand, next‐generation smart bone cement materials with properties such as biodegradability, immune responsiveness, and controlled release should be developed to enable dynamic regulation of the immune microenvironment and sustained support for tissue repair. Mechanistically, integrating techniques such as single‐cell sequencing and multi‐omics analyses may help elucidate the roles of additional cell types and signaling pathways in material‐mediated inflammation regulation, thereby constructing a more comprehensive immune regulation network for wound healing. Clinically, it is recommended to design prospective, multicenter, randomized controlled trials to evaluate the efficacy and safety of ALBC in various DFU types, across different wound stages and comorbid conditions. Combining ALBC with personalized therapeutic strategies—such as co‐administration with stem cells or natural immunomodulators—may enhance wound healing efficiency and therapeutic precision. Through the in‐depth integration of basic and clinical research, ALBC may ultimately progress from bench to bedside, emerging as a novel tool for precision immunotherapy in treating DFUs.

## 5 CONCLUSION

This study systematically revealed how ALBC significantly accelerated wound healing in DFUs by inhibiting NLRP3 inflammasome activation and promoting the polarization of macrophages from the pro‐inflammatory M1 phenotype to the anti‐inflammatory M2 phenotype. ALBC‐H exhibited pronounced anti‐inflammatory and pro‐healing effects both in vivo and in vitro, significantly reducing the expression of inflammatory cytokines, enhancing neovascularization and tissue regeneration, and demonstrating its potential to modulate macrophage polarization and alleviate inflammation in clinical samples. Although this study provided a novel therapeutic strategy for DFU wound healing and offered theoretical and experimental support for clinical application, several limitations remain. The study primarily used db/db mouse models, which may not fully replicate human disease conditions. In vitro experiments relied on a single RAW264.7 macrophage cell line, which may not capture the full heterogeneity of macrophage populations. In addition, the long‐term safety and potential side effects of ALBC require further evaluation. Future studies should validate the efficacy of ALBC across multiple animal models and investigate its potential applications in other types of chronic wounds. Moreover, multicenter clinical trials are needed to assess the safety and effectiveness of ALBC in DFU patients and explore possible synergistic mechanisms when combined with other therapeutic strategies.

## AUTHOR CONTRIBUTIONS

**Yi Zhang** and **Fusen Jia**: contributed equally to the study design, data collection, and analysis. **Yi Zhang**: performed the in vivo experiments, including the diabetic mouse model, while **Fusen Jia** carried out in vitro macrophage assays. **Ming Li**: assisted in the histological analyses and cytokine profiling. **Xin Tang** and **Fei Yang**: supervised the research, provided critical feedback on the experimental design, and co‐authored the manuscript. **Xin Tang** and **Fei Yang**: contributed equally as corresponding authors. All authors reviewed and approved the final manuscript.

## CONFLICT OF INTEREST STATEMENT

The authors declare no conflict of interest.

## Supporting information


**Table S1.** RT‐qPCR primer sequences (Mouse).
**Table S2.** Antibody manufacturer information.

## Data Availability

The data that support the findings of this study are available from the corresponding author upon reasonable request.
