# Supplementary material for: Engineering macrophage phenotype switching via nucleotide‐binding oligomerization domain‐like receptor protein 3 inflammasome inhibition: A translational approach using antibiotic cement for diabetic foot ulcers
Source: Bioeng Transl Med. 2025 Oct 15;10(6):e70073. doi: 10.1002/btm2.70073 (PMC12617559; doi:10.1002/btm2.70073)
Supplement: Supplementary file 1 — Table S1. RT‐qPCR primer sequences (Mouse). Table S2. Antibody manufacturer information. [file BTM2-10-e70073-s001.docx]

**Table S1. RT-qPCR primer sequences (Mouse).**

| **Gene** | **Forward Primer** |
| --- | --- |
| TNF-α | F:5'-CTGAACTTCGGGGTGATCGG-3' |
|  | R:5'-GGCTTGTCACTCGAATTTTGAGA-3' |
| iNOS | F:5'-ACATCGACCCGTCCACAGTAT-3' |
|  | R:5'-CAGAGGGGTAGGCTTGTCTC-3' |
| CD206 | F:5'-GAGGGAAGCGAGAGATTATGGA-3' |
|  | R:5'-GCCTGATGCCAGGTTAAAGCA-3' |
| CD163 | F:5'-AGTCCTGGATCATCTGTGACAAC-3' |
|  | R:5'-ACACGTCCAGAACAGTCTGTATG-3' |
| GAPDH | F:5'-CCAGTATGACTCCACTCACG-3′ |
|  | R:5'-GACTCCACGACATACTCAGC-3′ |

**Table S2. Antibody manufacturer information.**

| **Primary antibody** | **Catalog Number** | **Concentration** | **Manufacturer** |
| --- | --- | --- | --- |
| TNF-α | ab183218 | 1:1000 | Abcam |
| iNOS | ab178945 | 1:1000 | Abcam |
| CD206 | ab300621 | 1:1000 | Abcam |
| CD163 | ab182422 | 1:1000 | Abcam |
| pro-Caspase-1 | AF5418 | 1:1000 | Affinity Biosciences LTD |
| Cleaved-Caspase-1 (p20) | AF4005 | 1:500 | Affinity Biosciences LTD |
| GAPDH | ab9485 | 1:2500 | Abcam |
